# Learning curve of i-gel insertion in novices using a cumulative sum analysis

**DOI:** 10.1038/s41598-023-34152-5

**Published:** 2023-05-02

**Authors:** Toshiyuki Nakanishi, Seishi Sakamoto, Manabu Yoshimura, Koichi Fujiwara, Takashi Toriumi

**Affiliations:** 1 Department of Anesthesiology and Intensive Care Medicine, Nagoya City University Graduate School of Medical Sciences, 1 Kawasumi, Mizuho-Cho, Mizuho-Ku, Nagoya, Japan; 2 Department of Anesthesiology, Japan Community Healthcare Organization Tokuyama Central Hospital, Shunan, Japan; 3 Department of Materials Process Engineering, Nagoya University, Nagoya, Japan; 4 Department of Anesthesiology, Ube Industries Central Hospital, Ube, Japan; 5 Department of Anesthesiology, Nippon Kokan Fukuyama Hospital, Fukuyama, Japan

**Keywords:** Health care, Medical research

## Abstract

The i-gel, a popular second-generation supraglottic airway device, has been used in a variety of airway management situations, including as an alternative to tracheal intubation for general anesthesia, rescue in difficult airway settings, and out-of-hospital cardiac arrest resuscitation. We aimed to investigate the number of experiences needed to achieve a rapid, highly successful first attempt i-gel insertion in novices with a cumulative sum analysis. We also looked at how learning affected success rates, insertion time, and bleeding and reflex (limb movement, frowning face, or coughing) incidences. This prospective observational study included 15 novice residents from March 2017 to February 2018 in a tertiary teaching hospital. Finally, 13 residents with 35 [30–42] (median [interquartile range]) cases of i-gel insertion were analyzed. The cumulative sum analysis showed that 11 of 13 participants had an acceptable failure rate after 15 [8–20] cases. With increasing experience, success rate (*P* = 0.004), insertion time (*P* < 0.001), and incidence of bleeding (*P* = 0.006) all improved. However, the incidence of reflex did not change (*P* = 0.43). Based on our results, we suggest that 20 cases are preferable for novices to develop skills in using the i-gel in airway management.

## Introduction

The i-gel® (Intersurgical, Wokingham, UK) is a gel-like second-generation supraglottic airway device (SGA) with a gastric tube channel and no inflatable cuff. The i-gel has been used in a variety of airway management situations, including as an alternative to tracheal intubation for general anesthesia^1^, rescue in difficult airway settings^2^, and out-of-hospital cardiac arrest resuscitation^3^.

The i-gel has been reported to have a faster insertion time and a lower incidence of blood staining than other types of SGAs^4,5^. Furthermore, the largest multicenter prospective study examining 2049 i-gel insertions reported an overall first-time success rate as high as 93%, which was higher for novices than experienced anesthesiologists, implying a steep or absent learning curve^6^. However, early studies with novices revealed unsatisfactory first-time success rates ranging from 30 to 82.5%^7–9^. Thus, whether the i-gel is easy to insert for a novice remains unknown.

Cumulative sum (CUSUM) analysis is a statistical method used to evaluate learning curves to achieve proficiency with various procedures in medicine^10–12^. Several CUSUM-based studies in anesthesiology have shown that tracheal intubation requires 29–43 cases of experience for proficiency^10,13^. However, few studies have examined the number of experiences required for proficiency with SGA insertion, although we previously evaluated the learning curve of LMA® ProSeal™ (pLMA, Teleflex Incorporated, Wayne, PA, USA) insertion^14^. Therefore, the number of cases required for a novice to become proficient in i-gel insertion is unknown. Since i-gel is used in various settings^1–3^, establishing a target number of experiences for novices is crucial from an educational standpoint.

The purpose of this study was to investigate the learning curves of i-gel insertion in novice residents and the number of experiences required to achieve a rapid, highly successful first attempt insertion. We also examined the learning curves for success rates with a conventional definition, insertion time, and bleeding and reflex incidences during i-gel insertion.

## Methods

This single-center, prospective observational study was carried out from March 2017 to February 2018 after receiving approval from the Tokuyama Central Hospital Institutional Review Board (K231-20170111, approved on January 11, 2017). The Tokuyama Central Hospital Institutional Review Board waived patients’ consent in our tertiary teaching hospital (Tokuyama Central Hospital) because all patients were informed in advance that the residents perform the procedures under the supervision of the attending physicians. This study was registered prior to subjects’ enrollment at the University Hospital Medical Information (UMIN) Clinical Trials Registry (UMIN000020495, https://center6.umin.ac.jp/cgi-open-bin/ctr_e/ctr_view.cgi?recptno=R000023664, Principal investigator: T.N., Date of registration: January 8, 2016). The study was performed in accordance with the Declaration of Helsinki. This manuscript follows the STROBE guidelines.

We included novice residents with limited experience in airway management during their 1-month training in our anesthesiology department during their 2-year residency program immediately following graduation. Exclusion criteria were previous use of SGAs, including manikins, and < 80% use of i-gel among the first 20 cases of SGA insertion to ensure that the learning curve of i-gel was not affected. We gave the participating residents an i-gel lecture and showed them an instructional video on i-gel insertion technique (https://www.youtube.com/watch?v=YuG6k6ndBpM, viewed on September 29, 2022). We also took the i-gel and allowed the residents to freely hold and touch it. The study did not include predetermined manikin training. All residents who took part learned by observing experienced anesthesiologists (T.N., S.S., M.Y., and T.T.; > 1000 SGA insertions with ≥ 50 i-gel experience) insert i-gel into one to three patients.

Patients over the age of 18 years who were undergoing general anesthesia with SGAs were included in the study. Patients with a full stomach, a body mass index (BMI) > 35 kg m^−2^, gastroesophageal reflux, or an expected difficult airway were excluded. In the operating room, an electrocardiogram, pulse oximeter, and non-invasive blood pressure monitoring were started. After 3 min of pre-oxygenation, patients were given 2 μg kg^−1^ fentanyl and 1–2.5 mg kg^−1^ propofol intravenously, and mask ventilation with 5% sevoflurane was performed. Neuromuscular blocking drugs were not routinely used. The attending anesthesiologists determined the size of the i-gel based on the patient’s weight (size 3 for 30–60 kg and size 4 for 50–90 kg), height, and sex. The timing of i-gel insertion was determined by the attending anesthesiologists. Participated residents opened the patient’s mouth by themselves and inserted the i-gel with their other hand. An attending anesthesiologist could help with opening the patient’s mouth and jaw thrust, as well as provide verbal advice as needed.

Attending anesthesiologists documented success or failure, insertion time, ventilation quality, and bleeding and reflex during insertion. We defined a successful insertion as effective ventilation in a single attempt within 60 s, with the i-gel acting as a rescue. However, during a scheduled induction of general anesthesia, a quick and single-attempt placement of the device is not required because oxygenation is maintained as long as mask ventilation is maintained. Indeed, previous studies evaluating proficiency in airway procedures during anesthesia induction defined success as up to two attempts and within 120 s^13–15^. Thus, to compare with previous CUSUM-based studies, we also defined loose success criteria as one in which effective ventilation was obtained within 120 s and two attempts^13–15^. The insertion time was defined as the time between picking up the i-gel and observing the first upstroke of the capnogram^7,8,14^. Ventilation was graded as good (tidal volume ≥ 6 ml kg^−1^ and phase 3 of capnogram were observed), fair (tidal volume < 6 ml kg^−1^, lack of phase 3 of capnogram, or audible leak was observed), or failed (tidal volume or capnogram was not be observed). Bleeding on lips, tongue, and laryngopharynx was observed during insertion and after removal of the i-gel. Reflexes included limb movement, frowning face, or coughing during an i-gel insertion. If hypoxemia, moderate bleeding, or any other difficulties occurred during an i-gel insertion, the procedure was terminated at the discretion of the attending anesthesiologist and was considered a failure.

We designed our primary outcome to be the number of experiences required to gain proficiency with i-gel insertion, calculated by the CUSUM method. The secondary outcome was the success rate (with 60 s in a single attempt), the success rate with loose criteria (with 120 s in two attempts), insertion time, and bleeding and reflex incidences based on the number of experiences.

### Statistical analysis

Because it is a statistical method that focuses on the result rather than the process of performing procedural skills, the CUSUM analysis has been used to evaluate an individual’s procedural performance^10,13,14^. To conduct a CUSUM analysis, acceptable (p0) and unacceptable (p1) failure rates and type I and II errors (α and β) were set^10^.

Upper and lower decision limits (h1 and h0) were determined as follows:

$${\text{h1}} = {\text{a}}/\left( {{\text{P }} + {\text{ Q}}} \right)\;{\text{and}}\;{\text{ h}}0 = - {\text{b}}/\left( {{\text{P }} + {\text{ Q}}} \right),$$

where a = ln [(1 − β)/α], b = ln [(1 − α)/β] and

$${\text{P }} = {\text{ ln }}\left( {{\text{p1}}/{\text{p}}0} \right)\;{\text{and}}\;{\text{Q }} = {\text{ ln }}\left[ {\left( {{1 } - {\text{ p}}0} \right)/\left( {{1 } - {\text{ p1}}} \right)} \right].$$

CUSUM charts were created by plotting case numbers on the x-axis and CUSUM on the y-axis. When a successful attempt was recorded, the quantity S [Q/(P + Q)] was subtracted from the prior value, resulting in a downward trend. When an attempt failed, the quantity 1 − S was added to the previous value, resulting in an upward trend. If the line crossed the upper decision limit (h1) from below, the true failure rate was judged to be significantly higher than the unacceptable failure rate. If the line crossed the lower decision limit (h0) from above, the true failure rate was deemed not significantly different from the acceptable failure rate. If CUSUM remained within two boundary lines, the statistical inference could not be made.

To conduct CUSUM calculations, acceptable (p0) and unacceptable (p1) failure rates and type I and II errors (α and β) should be predefined^10^. Most previous reports examining the learning curve for anesthetic procedures conventionally designed the 20% acceptable failure rate, 40% unacceptable failure rate, 0.1 type I error, and 0.1 type II error^10,13,14,16^. We used the same parameters to conduct the CUSUM analysis. We recruited 15 novice residents because a similar number was used in previous studies^13,14^. In addition to the CUSUM chart, we created a chart depicting the cumulative success rate of each participant.

To evaluate the learning effects on success rate, insertion time, and incidences of bleeding and reflex, we stratified the numbers of i-gel insertions for each novice resident into four groups of 10 cases each (1–10, 11–20, 21–30, and ≥ 31 cases)^14^. Insertion time was also stratified into eight groups of 5 cases each (1–5, 6–10, 11–15, 16–20, 21–25, 26–30, 31–35, and ≥ 36 cases) to visualize in more detail. We used Fisher’s exact test to compare the four groups’ success rates and incidences of bleeding and reflexes. The Kruskal–Wallis test was used to compare insertion time. Bonferroni’s correction was used to adjust multiple comparisons.

The respective number of procedures performed until h0 was crossed and insertion time are presented as median [interquartile range]. Success rates and incidences of bleeding and reflex are presented as numbers (proportion). Individual novice residents were represented by randomly assigned capital letters. R software (version 3.6.3, R Foundation for Statistical Computing, Vienna, Austria) was used for all the statistical analyses. Moreover, Microsoft® Excel for Mac (version 16.65, Microsoft Corp., Redmond, WA, USA) was used to construct the CUSUM chart. *P* values of < 0.05 were deemed statistically significant.

## Results

Fifteen novice residents were included, with two residents being excluded due to the dearth of i-gel cases among their first 20 cases (10 cases each). Finally, we analyzed 13 residents who had 35 [30–42] i-gel insertions. Overall, 464 patients underwent i-gel insertion, with novice residents successfully ventilating 412 patients (89%) via i-gel. The remaining 52 patients were successfully ventilated with i-gel by supervising anesthesiologists. The patients were 69 [56–78] years old, 58% female, 157 [150–165] cm tall, 55 [47–64] kg of weight, and 22 [20–24] kg m^−2^ of BMI.

**Figure 1:**
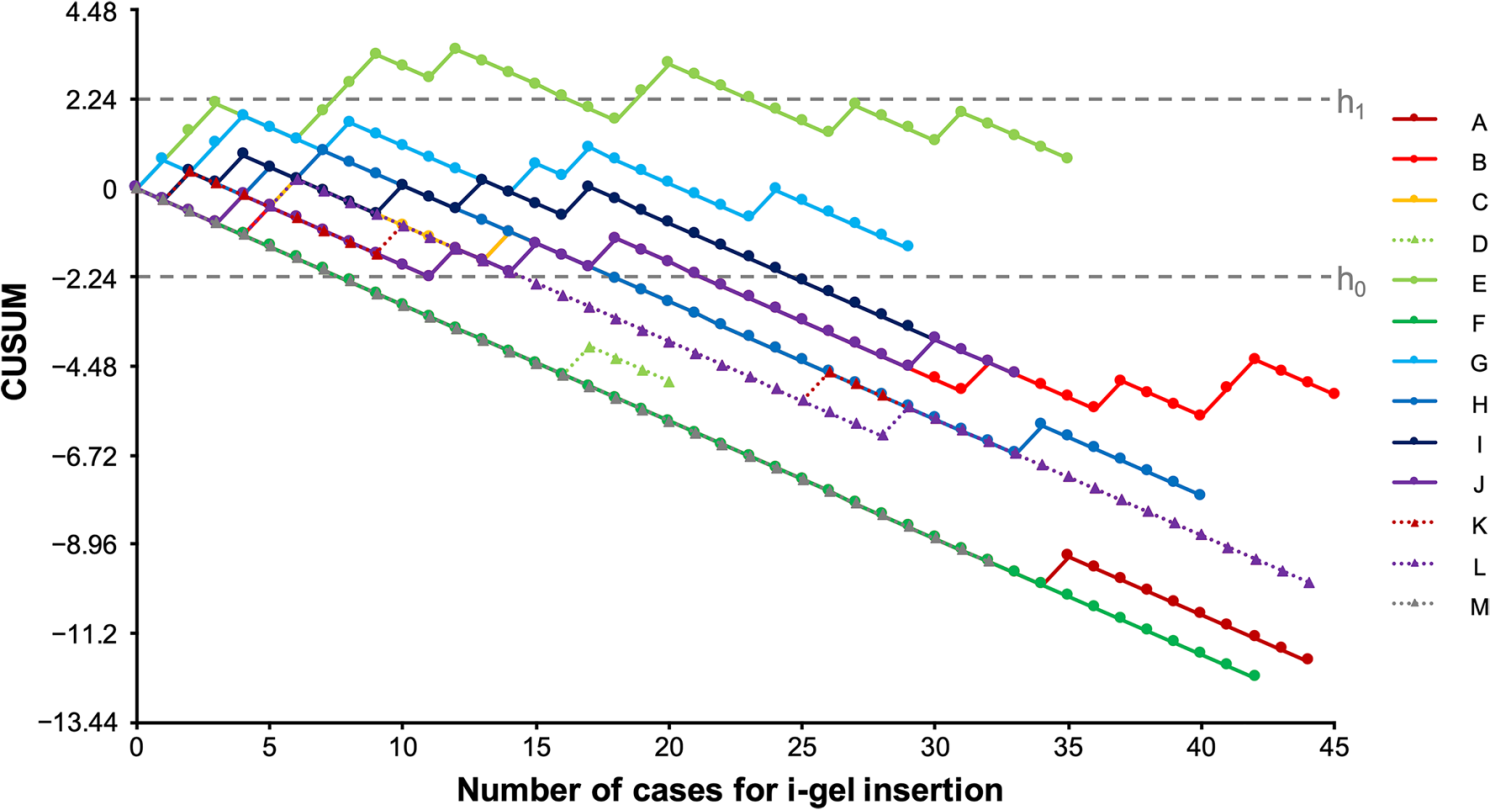
Cumulative sum chart of individual successful i-gel insertion. Lines A–M represent the cumulative sum of successful i-gel insertion performed by individual residents. The upper and lower decision limits of 2.24 and − 2.24 are represented by lines h1 and h0, respectively. The Y-axis values are multiples of h1 and h0. *CUSUM* cumulative sum.

**Figure 2:**
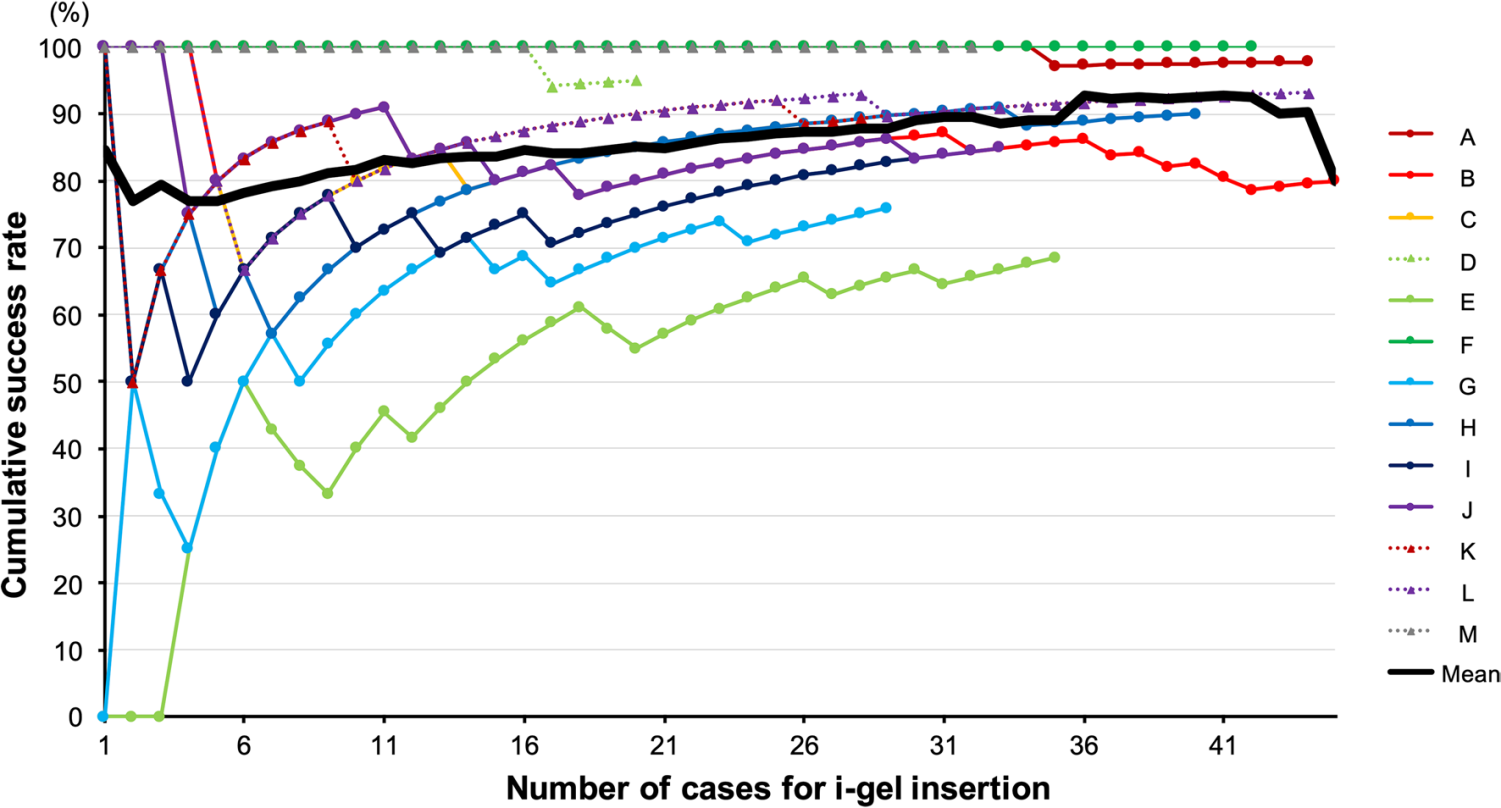
Individual and mean cumulative success rates of i-gel insertion. Lines A–M represent the cumulative success rates of i-gel insertion performed by individual residents. Mean values of success rate are represented in a bold black line.

**Figure 3:**
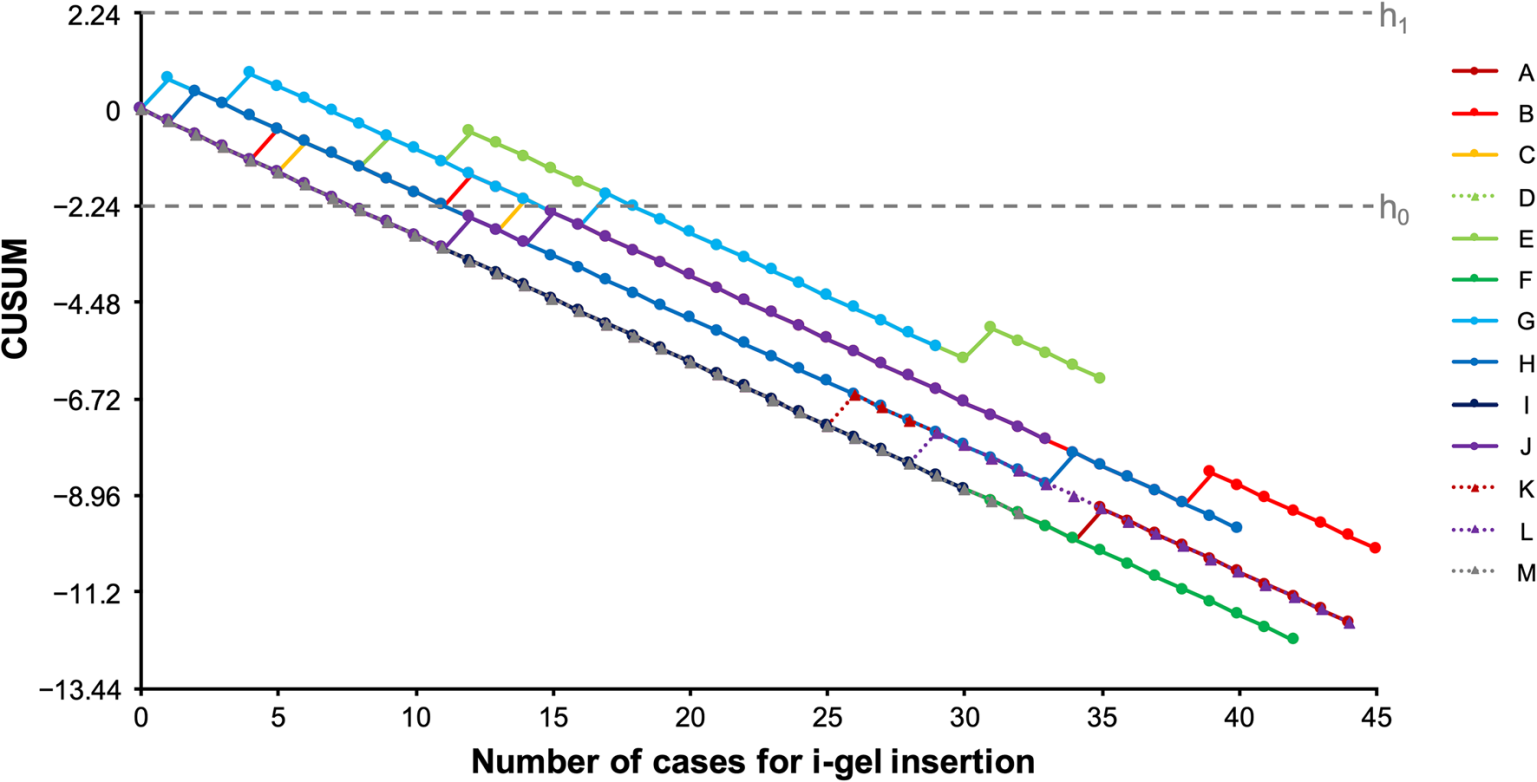
Cumulative sum chart of successful i-gel insertion within two attempts and ≤ 120 s duration. Lines A–M represent the cumulative sum of successful i-gel insertion performed by individual novice residents. The upper and lower decision limits of 2.24 and − 2.24 are represented by lines h1 and h0, respectively. The Y-axis values are multiples of h1 and h0. *CUSUM* cumulative sum.

According to CUSUM analysis, 11 of 13 participants had an acceptable failure rate after 15 [8–20] cases of i-gel insertion with ≤ 60 s and in a single attempt (Table 1 and Fig. 1). Figure 2 depicts the cumulative success rate of each participant. In CUSUM analysis, all 13 residents achieved an acceptable failure rate with 8 [8–12] cases in the loose criteria of success rate within two attempts and up to 120 s (Fig. 3).

**Table 1:** Success rates and number of attempts to cross h0 in cumulative sum analysis for individual resident.

Resident	Success/attempt	Success rate % (95% CI)	Attempts to cross h0
A	43/44	98 (88–100)	8
B	36/45	80 (65–90)	22
C	25/28	89 (72–98)	18
D	19/20	95 (75–100)	8
E	24/35	69 (51–83)	Did not cross
F	42/42	100 (92–100)	8
G	22/29	76 (57–90)	Did not cross
H	36/40	90 (76–97)	18
I	25/30	83 (65–94)	25
J	28/33	85 (68–95)	22
K	39/42	93 (81–99)	15
L	41/44	93 (81–99)	15
M	32/32	100 (89–100)	8
Total	412/464	89 (86–92)	15 [8–20]

Capital letters A–M indicate each resident. Data are expressed as number, proportion (95% confidence interval), or median [interquartile range].

**Figure 4:**
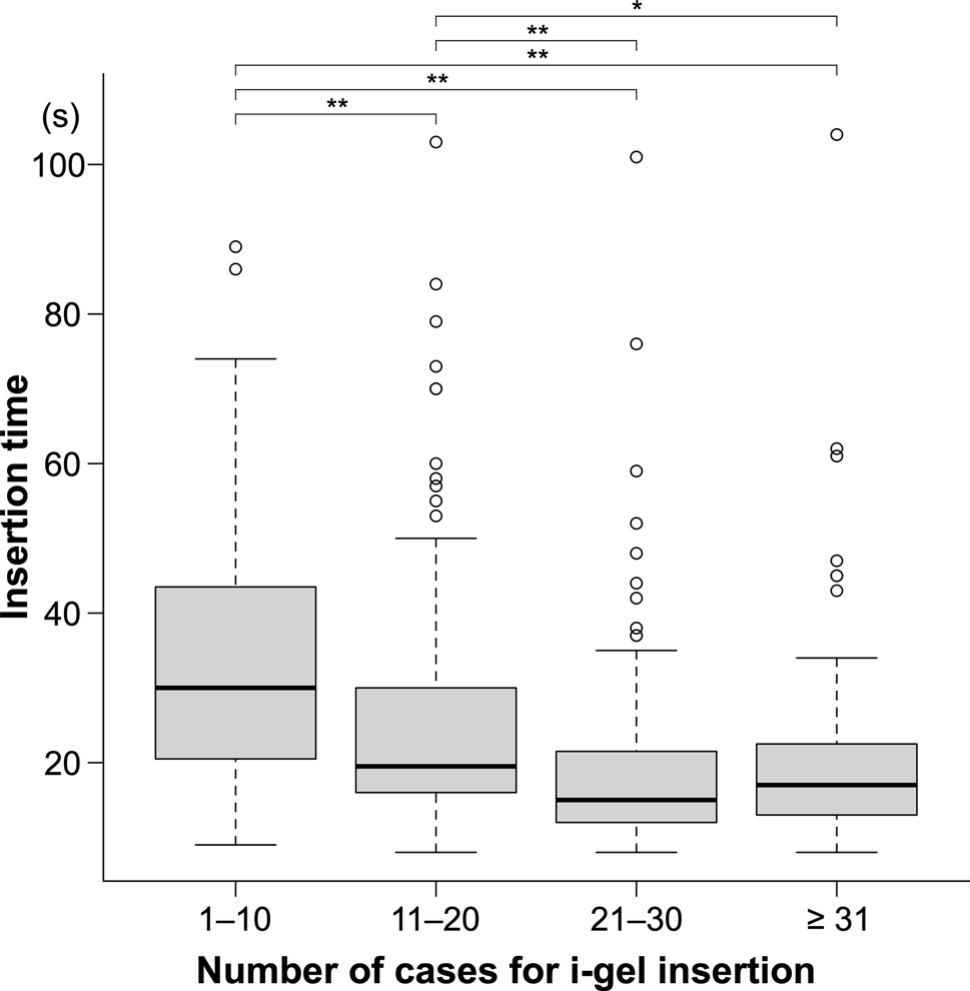
Insertion time in the stratified four groups according to each 10 cases. The box and whisker plots show the time for successful i-gel insertion divided by the number of cases. The boxes’ lower and upper edges represent the 25th and 75th percentiles, respectively. The medians are represented by the bold horizontal lines that run across the boxes. The whiskers represent the lowest and highest values from the 25th and 75th percentiles within a 1.5-box length. Outliers (between 1.5 and 3 box lengths from the 75th percentile) are shown as circles. *P < 0.05; **P < 0.001. *P* values were adjusted using the Bonferroni method.

With increasing experience, the first success rate (*P* = 0.004), insertion time (*P* < 0.001), and incidence of bleeding (*P* = 0.006) all improved (Table 2 and Fig. 4). In particular, the first success rate significantly increased between the 1–10 cases and the 21–30 cases (*P* = 0.004, with Bonferroni adjustment). Furthermore, the incidence of bleeding significantly reduced between 11–20 and 21–30 cases (*P* = 0.02, with Bonferroni adjustment). However, even in the 1–10 cases, the success rate within two attempts and up to 120 s was as high as 95% and showed no significant change with increased experience (Table 2). The frequency of reflexes did not change significantly with experience (Table 2).

**Table 2:** Learning effects of i-gel insertion on success rate, insertion time, and incidence of bleeding and reflex.

	1–10 cases (n = 130)	11–20 cases (n = 130)	21–30 cases (n = 117)	≥ 31 cases (n = 87)	*P* value
Success rate	106 (82%)	115 (89%)	112 (96%)	79 (91%)	0.004^a^
Success rate within 120 s and two attempts	123 (95%)	124 (95%)	115 (98%)	83 (95%)	0.46
Incidence of bleeding	3 (2%)	9 (7%)	0 (0%)	1 (1%)	0.006^b^
Incidence of reflex	4 (3%)	3 (2%)	2 (2%)	0 (0%)	0.43

Data are expressed as number (proportion). *P* values are from the Fisher’s exact test.

^a^*P* = 0.004 between 1–10 cases and 21–30 cases with Bonferroni adjustment.

^b^*P* = 0.02 between 11–20 cases and 21–30 cases with Bonferroni adjustment.

**Figure 5:**
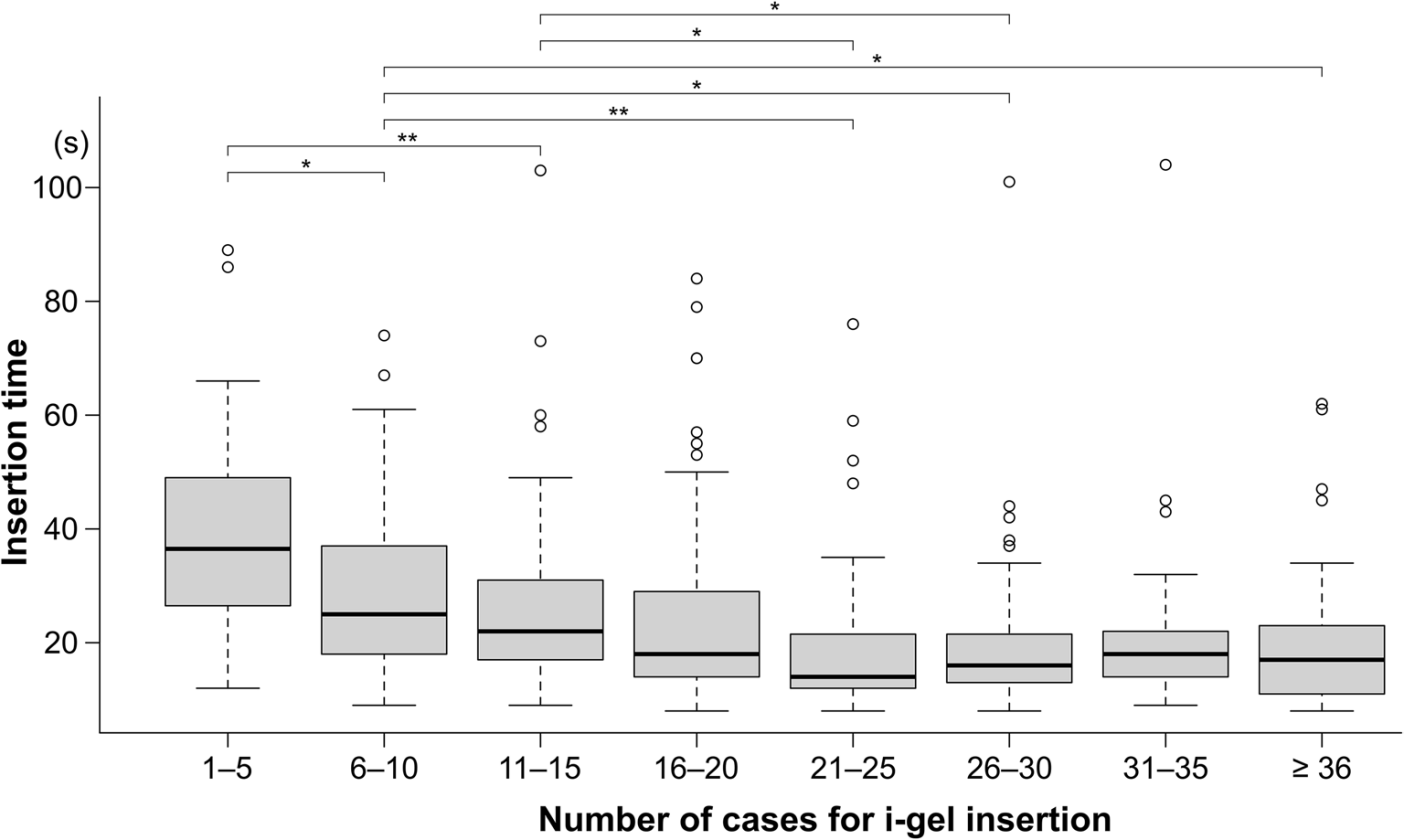
Insertion time in the stratified eight groups according to each five cases. The box and whisker plots show the time for successful i-gel insertion divided by the number of cases. The boxes’ lower and upper edges represent the 25th and 75th percentiles, respectively. The medians are represented by the bold horizontal lines that run across the boxes. The whiskers represent the lowest and highest values from the 25th and 75th percentiles within a 1.5-box length. Outliers (between 1.5 and 3 box lengths from the 75th percentile) are shown as circles. *P < 0.05; **P < 0.001. *P* values were adjusted using the Bonferroni method. *P* values were < 0.001 between 1–5 cases and ≥ 16 cases, although not shown in the figure.

With statistical significance, the insertion time was reduced sequentially from 1–10 cases to 21–30 cases (Fig. 3). There was no difference in insertion time between the 21–30 and ≥ 31 cases (*P* > 0.99). Figure 5 depicts the insertion time in greater detail for the eight groups, demonstrating that the insertion time decreased with experience from the first to the 21–25 cases.

## Discussion

In this prospective observational study, 13 novice residents recorded the insertion properties of the i-gel in a total of 464 patients. We discovered that 15 [8–20] cases were required to achieve a 20% acceptable failure rate for successful i-gel insertion in a single attempt within 60 s using a CUSUM analysis.

The required number for proficiency in i-gel was less than previously reported for tracheal intubation, which required a median of 29–57 cases^10,13,15^, or mask ventilation, which required a median of 25 cases^13^. The steep learning curve is a major advantage because the i-gel is also used as a rescue device in a difficult airway setting^2^ and resuscitation^3^. However, the gradual improvement in success rate up to the 20th case observed in our study may indicate the existence of a certain learning curve in the early stages of experience. Based on our results that 15 [8–20] cases of experience were needed for a rapid and highly successful first attempt of i-gel insertion, we propose that at least 20 cases of experience are required for novices.

Two of the thirteen residents did not meet the proficiency criteria. In addition to individual differences in learning speed, numerous early failures may have contributed to the failure to achieve proficiency. On the CUSUM chart, more successes are required to cross the h0 line when there are more failures in the early phase^10^. Since both residents had decreasing CUSUM values at the end of the study, they may have reached the h0 line with increasing experience. Future research on the interventions that steepen the learning curve is warranted.

Even in the first ten cases, with the loose criteria of successful insertion within 120 s up to two attempts, the success rate was as high as 95%, indicating no improvement based on experience (Table 2). Thus, when using the loose success criteria designed for use in scheduled airway management, our results show that the learning curve of i-gel insertion is extremely steep or absent. As a result, training in i-gel insertion may not be required for those who are not involved in emergency airway management.

We discovered that the insertion time of i-gel decreased significantly in the first 15 cases and converged to 15–20 s in novice residents. In previous studies, i-gel insertion time was reported to be 17.5–28 s in novices^7,8^ and 15–17.5 s in experienced anesthesiologists^17,18^. Taken together, our findings suggest that 15 cases were required for novices to insert an i-gel in 15–20 s, comparable to experienced anesthesiologists.

The overall incidence of bleeding in our study was 3%, which was lower than in a previous large study (3.9%, 79/2049)^6^. We found that the incidence of bleeding was highest at 7% in 11–20 cases and then decreased significantly in the subsequent 21–30 cases (Table 2). One possible explanation for the high incidence of bleeding in 11–20 cases is that the novices’ technique became cruder due to their familiarity after 10 cases of experience. The overall incidence of reflex was 1.9%, and did not vary with experience. Thus, our study demonstrated that novices could insert i-gel with low complication rates comparable to experienced anesthesiologists.

We compared the current results of i-gel to our previous study evaluating pLMA insertion in the same setting^14^. The success rates of i-gel in a single attempt within 60 s were consistently higher than those of pLMA (67%, 75%, 84%, and 87% in 1–10, 11–20, 21–30, and ≥ 31 cases, respectively)^14^. The i-gel also showed higher success rates within two attempts and ≤ 120 s than pLMA (76%, 86%, 91%, and 93%)^14^. Moreover, i-gel had a shorter insertion time and fewer incidences of bleeding and reflex than pLMA^14^. As a result, the current study suggests that i-gel may be easier and safer for novices to secure the airway than pLMA.

This study has several noteworthy strengths. First, we assessed the learning curve for i-gel insertion on actual patients rather than manikins. Second, we evaluated the i-gel, which has been used in a variety of clinical settings since its introduction^1–3^. As a result, our findings may be useful in anesthesiologists’, emergency physicians’, and paramedics’ training and education. Finally, we performed a CUSUM analysis using the same parameters as the previous reports^10,13,14^. This allowed us to compare and interpret the characteristics of i-gel insertion with the other airway procedures during the early stages of learning.

Our research has some limitations. For starters, this was a single-center study. Other settings, such as other regions, participants (both residents and patients), or outside of operating rooms, may yield different results than ours. Second, patients with BMI > 35 and those expected to have a difficult airway were excluded. Learning curves of i-gel insertion may also differ in such patients with difficult airways. Finally, we did not conduct simulation training with a manikin before enrolling the residents in the study. Although our results were comparable or even superior to previous studies in terms of success rate, insertion time, and bleeding complications^6–8^, it remains to be seen whether manikin training affects the learning curve.

In conclusion, 15 [8–20] cases were required to achieve proficiency in novice residents for successful i-gel insertion with a single attempt and ≤ 60 s duration. We, therefore, suggest that 20 cases are ideal for novices to practice inserting the i-gel in both scheduled and emergency airway settings.

## Data Availability

The data that support the findings of this study are available from the corresponding author, T.N., upon reasonable request.

## References

[1] Uppal V, Fletcher G, Kinsella J (2009). Comparison of the i-gel with the cuffed tracheal tube during pressure-controlled ventilation. Br J Anaesth.

[2] Kleine-Brueggeney M, Theiler L, Urwyler N, Vogt A, Greif R (2011). Randomized trial comparing the i-gel™ and Magill tracheal tube with the single-use ILMA™ and ILMA™ tracheal tube for fibreoptic-guided intubation in anaesthetized patients with a predicted difficult airway. Br J Anaesth.

[3] Benger JR (2018). Effect of a strategy of a supraglottic airway device vs tracheal intubation during out-of-hospital cardiac arrest on functional outcome. JAMA.

[4] de Montblanc J, Ruscio L, Mazoit JX, Benhamou D (2014). A systematic review and meta-analysis of the i-gel® vs laryngeal mask airway in adults. Anaesthesia.

[5] Park SK, Choi GJ, Choi YS, Ahn EJ, Kang H (2015). Comparison of the I-gel and the laryngeal mask airway proseal during general anesthesia: A systematic review and meta-analysis. PLoS ONE.

[6] Theiler L (2012). I-gel™ supraglottic airway in clinical practice: A prospective observational multicentre study. Br J Anaesth.

[7] Wharton NM (2008). I-gel insertion by novices in manikins and patients. Anaesthesia.

[8] Ragazzi R, Finessi L, Farinelli I, Alvisi R, Volta CA (2012). LMA supreme™ vs i-gel™—A comparison of insertion success in novices. Anaesthesia.

[9] Hattori K (2016). Muscle relaxant facilitates i-gel insertion by novice doctors: A prospective randomized controlled trial. J Clin Anesth.

[10] Filho DO, Rodrigues G (2002). The construction of learning curves for basic skills in anesthetic procedures: An application for the cumulative sum method. Anesth Analg.

[11] Holzhey DM, Seeburger J, Misfeld M, Borger MA, Mohr FW (2013). Learning minimally invasive mitral valve surgery: A cumulative sum sequential probability analysis of 3895 operations from a single high-volume center. Circulation.

[12] van der Poel MJ (2016). Outcome and learning curve in 159 consecutive patients undergoing total laparoscopic hemihepatectomy. JAMA Surg.

[13] Komatsu R (2010). Learning curves for bag-and-mask ventilation and orotracheal intubation: An application of the cumulative sum method. Anesthesiology.

[14] Nakanishi T, Sakamoto S, Yoshimura M, Toriumi T (2020). A learning curve of LMA® ProSeal™ insertion: A prospective analysis of cumulative sum method. J Anesth.

[15] Konrad C, Schüpfer G, Wietlisbach M, Gerber H (1998). Learning manual skills in anesthesiology: Is there a recommended number of cases for anesthetic procedures?. Anesth Analg.

[16] Kestin IG (1995). A statistical approach to measuring the competence of anaesthetic trainees at practical procedures. Br J Anaesth.

[17] Francksen H (2009). A comparison of the i-gel™ with the LMA-Unique™ in non-paralysed anaesthetised adult patients. Anaesthesia.

[18] Lee JS (2020). Prospective, randomized comparison of the i-gel and the self-pressurized air-Q intubating laryngeal airway in elderly anesthetized patients. Anesth Analg.

